# Free triiodothyronine (T3) is negatively associated with fasting ghrelin serum levels in a population sample of euthyroid subjects

**DOI:** 10.1007/s40618-021-01578-5

**Published:** 2021-04-21

**Authors:** D. A. Wittekind, J. Kratzsch, R. Mergl, R. Baber, V. Witte, A. Villringer, M. Kluge

**Affiliations:** 1 Department of Psychiatry and Psychotherapy, University of Leipzig, Semmelweisstrasse 10, 04103 Leipzig, Germany; 2 Institute of Laboratory Medicine, Clinical Chemistry and Molecular Diagnostics, University of Leipzig, Leipzig, Germany; 3 Institute of Psychology, Universität der Bundeswehr München, Neubiberg, Germany; 4 Department of Neurology, Max Planck Institute for Cognitive and Brain Sciences, Leipzig, Germany; 5 LIFE, Leipzig Research Center for Civilization Diseases, University of Leipzig, Philipp-Rosenthal-Strasse 27, 04103 Leipzig, Germany

**Keywords:** Ghrelin, Thyroid, Metabolism, Iodothyronine deiodinase, Homeostasis

## Abstract

**Purpose:**

Ghrelin is an orexigenic peptide hormone secreted in times of stress and hunger. It is deeply involved in the regulation of metabolism and energy homeostasis, promoting energy intake and inhibiting energy expenditure on a metabolic level. In this regard, it has in many ways antagonistic effect on the thyroid hormones, which increase metabolism and thus energy expenditure. While there is reasonable evidence of a negative association between ghrelin and hormones of the hypothalamic-pituitary-thyroid (HPT-) axis from studies in patients with thyroid dysfunction and small intervention studies, large-scale studies in healthy subjects are lacking. Therefore, we studied the relationship between total ghrelin serum levels and serum levels of the thyroid hormones in a large sample of euthyroid subjects.

**Methods:**

Total ghrelin, thyroid-stimulating hormone (TSH), free thyroxine (fT4) and free triiodothyronine (fT3) were determined after an overnight fast in 1666 subjects participating in a population-based cross-sectional study (‘LIFE’) including 10,000 adults. 1012 subjects were included in this analysis. Multiple linear regression analyses were performed.

**Results:**

FT3 was negatively associated with serum ghrelin; total sample: *β* = − 0.0001, *p* < 0.001; men: *β* = − 0.0002, *p* = 0.013; women: *β* = − 0.0001, *p* = 0.010, adjusted for age, BMI, alcohol consumption, serum levels of TSH and fT4 and smoking status. No associations were found between ghrelin serum levels and serum levels of fT4 or TSH.

**Conclusion:**

This is to date the largest study investigating the relationship between total serum ghrelin and thyroid hormones. The results point to a complex interaction and should initiate further research.

**Supplementary Information:**

The online version contains supplementary material available at 10.1007/s40618-021-01578-5.

## Introduction

Ghrelin is a 28-amino-acid peptide hormone that is predominantly synthesized in the stomach [1]. Ghrelin’s receptor, the G-protein-coupled Growth-Hormone-Secretagogue-Receptor (GHRS-1a) is widely expressed in a variety of tissues, but especially in endocrine tissue (e.g. thyroid) and the central nervous system [2]. Ghrelin binds to it after being acylated by the Ghrelin-O-acyl-transferase (GOAT) [3, 4]. Ghrelin is the only known peripheral orexigenic hormone which is secreted in times of hunger and when anticipating a meal [5, 6], promoting food intake and weight gain [7]. It is involved in glucose metabolism, suppressing pancreatic insulin secretion [8] and promoting hepatic secretion of insulin-like-growth-factor 1 and glucagon [9, 10]. In addition, ghrelin has been shown to decrease thermogenesis and energy expenditure [11, 12]. Combined, ghrelin acts toward a positive energy balance by increasing energy intake and decreasing energy expenditure. In line with this, ghrelin serum levels are elevated in underweight humans like in anorexia nervosa [13] and suppressed in obese individuals [14]. Furthermore, there is general agreement that ghrelin affects several endocrine axes: it exerts stimulatory effects on the hypothalamic–pituitary–adrenal axis (HPA) and the somatotropic axes [6, 15] and inhibitory effects on the hypothalamic–pituitary–gonadal (HPG) axis [6, 16]. In contrast, ghrelin’s effect on the hypothalamus-pituitary-thyroid (HPT) axis has not been acknowledged in the same way [6] although there is good evidence for an interplay between the ghrelin system and HPT axis [17, 18], with a majority of the evidence pointing to a negative association between ghrelin and thyroid hormones. For example, the administration of ghrelin suppressed TSH in animals [17] and humans [18].

Such an association is plausible since thyroid hormones exert to some extent opposing effects in the organism: they promote a negative energy balance by increasing metabolism and thermogenesis, thereby also increasing energy expenditure [19–21]. Thyroid hormones also play a key role in the maturation and differentiation of almost all tissues, including brain, liver, heart and muscle [22, 23]. Pituitary thyroid-stimulating-hormone (TSH) stimulates the thyroid gland to produce thyroxine (T4) and to a lesser extent triiodthyronine (T3), which is about 4 times more biologically active than T4. The largest proportion of T3, however, is produced from T4 by mainly extra-thyroidal deiodinisation by a family of specialized enzymes, the deiodinases [23, 24].

Most studies have so far focused on patients suffering from thyroid dysfunction (e.g. [25, 26]). Interventional studies in healthy subjects, investigating ghrelin’s effect on thyroid hormones and vice versa, were done in comparatively small samples [18, 27]. A large-scale study investigating the relationship between ghrelin serum levels and thyroid hormones is lacking. Therefore, we studied this relationship in a large, population-based sample of currently euthyroid subjects. We hypothesized that ghrelin serum levels are negatively associated with thyroid hormones.

## Methods

### Study design and subjects

Subjects were recruited in the context of the LIFE-Adult-Study (Leipzig Research Center for Civilization Diseases), a population-based cohort study with 10,000 participating adults mainly aged 40–79 years. The sample was randomly recruited in the city of Leipzig in Germany (for details of the study design see Loeffler 2015 [28]). Blood samples were collected after an overnight fast including abstinence from smoking between 07:30 and 10:30 h. The blood samples have been immediately processed by the team of the LIFE pre-analytical laboratory which is part of the Leipzig Medical Biobank and send directly to the Institute of Laboratory Medicine, Clinical Chemistry, and Molecular Diagnostics (ILM) where direct analysis were carried out (fT3, fT4, TSH). Additional samples were frozen at -80 °C until usage for ghrelin measurements.

When selecting study participants for ghrelin measurements, subjects with a history of stroke, multiple sclerosis or epilepsy were excluded. Total ghrelin was measured in 1666 subjects. From these, 151 subjects were excluded due to an incomplete data set, i.e. when any of the relevant variables was missing (e.g. ghrelin level, BMI, thyroid hormone levels, etc.). Subjects were excluded from the analysis if any of the thyroid hormones were not in the reference range (TSH 0.4–3.77 mU/l; fT4 12.8–20.4 pmol/l; fT3 3.1–6.79 pmol/l) [29, 30]. 295 subjects showed thyroid hormone levels outside of the reference range (for details see supplementary Table 1). A relatively high percentage of subjects showed thyroid hormone levels outside the reference range (295/1515 ≙ 19.5%). Of these, TSH was altered in 123 subjects (8.1%). 172 subjects (11.4%) showed alterations only in fT3 and/or fT4 with TSH in the reference range. Alterations only in the free thyroid hormones, but not in TSH can be caused by several factors like altered levels of serum binding proteins [31] or displacement of free hormones from protein binding by certain drugs like furosemide, aspirin, non-steroidal anti-inflammatory agents or heparin [31, 32]. Furthermore, other non-thyroidal disease states like chronic liver and renal disease [31, 33], familial dysalbuminaemic hyperthyroxinaemia and in rare cases assay interference may lead to altered free thyroid hormone levels in commercial assays [31]. Subjects with a history of thyroid disease or current (self-) medication with thyroid-relevant medication were also excluded. Overall, 208 (17.0%) further subjects had a history of thyroid disease. This high prevalence is in line with existing epidemiologic studies from Germany which estimated the prevalence of thyroid diseases at about 20–30% [34] with as many as 33% showing abnormal ultra-sound findings [34]. We interpret the high number of subjects with thyroid hormone levels outside of the reference range as a combination of the high prevalence of thyroid disease in the general population with the other factors discussed above. From the remaining individuals, nobody had current medication with thyroid relevant drugs, resulting in a study population of 1012 subjects (659 men, 353 women). Subjects with a gastro-intestinal pathology such as stomach/duodenal ulcer or inflammatory bowel disease (IBD) in the last 12 months were rare (0.4% and 0.45%, respectively) and were not excluded from the analysis, due to the low case number and the fact that these subjects were already in treatment for these conditions. All participants gave written informed consent to take part in the study. The procedures were conducted according to the Declaration of Helsinki and approved by the ethics committee of the University of Leipzig (registration-number: 263-2009-14122009).

### Ghrelin measurements

All ghrelin measurements were done in serum by the use of a radioimmunoassay for total ghrelin (Mediagnost, Reutlingen Germany). Samples were not pre-treated with enzyme inhibitors or acidification. Due to this, only total ghrelin was measured, as it is much more stable than acyl-ghrelin. Sensitivity of the assay was 0.04 ng/mL, mean intra-assay coefficients of variation were 2.7–4.3%; interassay coefficients of variation were between 6.9 and 9.2% for the mean expected range of clinical data around 0.88 and 0.97 ng/mL.

### TSH, fT4 and fT3 measurements

Parameters of thyroid function (TSH, FT3; FT4) were measured by electrochemiluminescence assays (ECLIA) via Cobas 601 or 801 (Roche Diagnostics, Germany). Quality control (QC) data were calculated for 10 QC cycles with 15–110 runs per cycle over a representative range of 4 months over 4 years. The mean interassay coefficient of variation of the three measured biomarkers ranged between 2.25% and 3.11%, the mean deviation from the target value was between 3.33 and 4.82%.

### Acquisition of data on tobacco and alcohol consumption

Tobacco consumption was assessed solely via self-administered questionnaire and interview. No biological verification method was used (like measurement of carbon monoxide in breath). Subjects were grouped into three categories: active smoker, former smoker and never-smoker. Active smokers were considered all those participants who had smoked regularly for at least 6 months consecutively in their lifetime and at least occasionally at the time of examination. Subjects who had smoked continuously for more than 6 months during their lifetime, but were not smoking at the time of assessment, were defined as former smokers. Cigarette equivalents (number per day) were calculated as follows: one cigarette equivalent was defined as 1 g of tobacco, 1 cigar was defined as 4 g of tobacco (i.e. 4 cigarette equivalents), 1 pipe as 3 g of tobacco (i.e. 3 cigarette equivalents), 1 cigarillo as 2 g of tobacco (i.e. 2 cigarette equivalents). For further information see Latza and others 2005 [35].

Frequency and amount of consumption of alcoholic beverages (i.e. beer/wine/spirits) during the last 12 months were semiquantitatively assessed using a self-administered food frequency and alcohol questionnaire (FFQ). Possible answers for the frequency of alcohol consumption were “multiple times a day”, “daily”, “multiple times a week”, “once a week”, “two to three times a month”, “once a month or rarer” or “almost never”. In addition, the amount of beverage consumption was assessed by defined categories. From the amount and frequency of alcoholic beverage as well as the average alcohol content of different beverages, the average consumption of pure alcohol (g/day) was calculated.

### Assessment of BMI

The BMI is defined as the body weight divided by the square of the body height (kg/m^2^). Body weight was measured with an electronic scale (SECA 701, Seca GmbH & Co KG) with a precision of 0.01 kg, height using a stadiometer (SECA 240) to the nearest 0.1 cm by trained staff according to standardized protocols.

### Statistical analysis

A multivariate linear regression analysis was performed. Presuming an effect of ghrelin on TSH/fT4/fT3 levels, the independent variable was ghrelin and the dependent variable was TSH/fT4/fT3 levels. The analysis was adjusted for age (continuous variable), sex (categorical variable; reference category: male), BMI (continuous variable) and TSH/fT4/fT3 levels respectively. In addition, the analysis was adjusted for smoking status (categorical variable; reference category: non-smokers) and alcohol consumption (continuous variable) both variables being associated with ghrelin levels [36, 37]. Regression coefficients (*β*) with 95% confidence intervals (CI) and corresponding *p* values were calculated. In addition, standardized regression coefficients are supplied to represent the effects of variables independent of units. The same was true regarding the proportion of variance accounted for by the regression model (*R*^2^).

Furthermore, extreme-group comparisons were performed, i.e. Mann–Whitney U tests were used to test for statistical differences regarding FT3 levels between the highest and lowest quartile according to ghrelin serum levels for the total sample, but also for men and women separately.

The SPSS version 24.0 was used for the statistical analyses and the significance level *α* = 0.05 was chosen. All statistical tests were two-sided. Where appropriate, data are presented as mean (standard deviation) throughout.

## Results

### Clinical data

Description of the study sample can be found in Table 1.

**Table 1:** Description of the sample

Variable	Total sample (*n* = 1012)	Males (*n* = 659) (65.1%)	Females (*n* = 353) (34.9%)	*p*-value
Ghrelin serum concentration, pg/ml, mean (SD)	893.26 (391.55)	814.81 (308.09)	1039.72 (479.43)	< 0.001***^a^
Age, years, mean (SD)	57.55 (15.23)	57.56 (15.48)	57.55 (14.78)	0.51^a^
BMI, kg/m^2^, mean (SD)	27.09 (4.35)	27.32 (3.99)	26.65 (4.92)	< 0.001***^a^
Alcohol consumption, g/day, mean (SD)	13.98 (18.14)	18.50 (20.18)	5.53 (8.57)	< 0.001***^a^
Non-smokers (%)	853 (84.3%)	544 (82.5%)	309 (87.5%)	0.04*^b^
Active smokers (%)	159 (15.7%)	115 (17.5%)	44 (12.5%)	–
TSH concentration, mU/l, mean (SD)	1.60 (0.72)	1.59 (0.70)	1.64 (0.74)	0.43^a^
FT3 concentration, mU/l, mean (SD)	5.08 (0.56)	5.21 (0.54)	4.83 (0.52)	< 0.001***^c^
FT4 concentration, mU/l, mean (SD)	15.98 (1.66)	16.05 (1.67)	15.84 (1.65)	0.08^a^

*SD* Standard Deviation

*p < 0.05; **p < 0.01; ***p < 0.001

^a^Due to non-normal distribution of the dependent variables (Kolmogorov–Smirnov-Test: *p* < 0.05), a Mann–Whitney U test was performed to compute p values regarding ghrelin serum concentrations (*Z* = − 8.73), age (*Z* = − 0.66), BMI scores (*Z* = − 3.30), alcohol consumption (*Z* = − 12.53), TSH concentrations (*Z* = − 0.79) and FT4 concentrations (*Z* = − 1.78)

^b^A chi-square test for a two-by-two cross table (*χ*^2^ = 4.32; df = 1) was applied

^c^A t test for independent sample comparison (*t* = 10.64; df = 1,1010) was computed

### Association between thyroid hormones and ghrelin serum levels

**Fig. 1:**
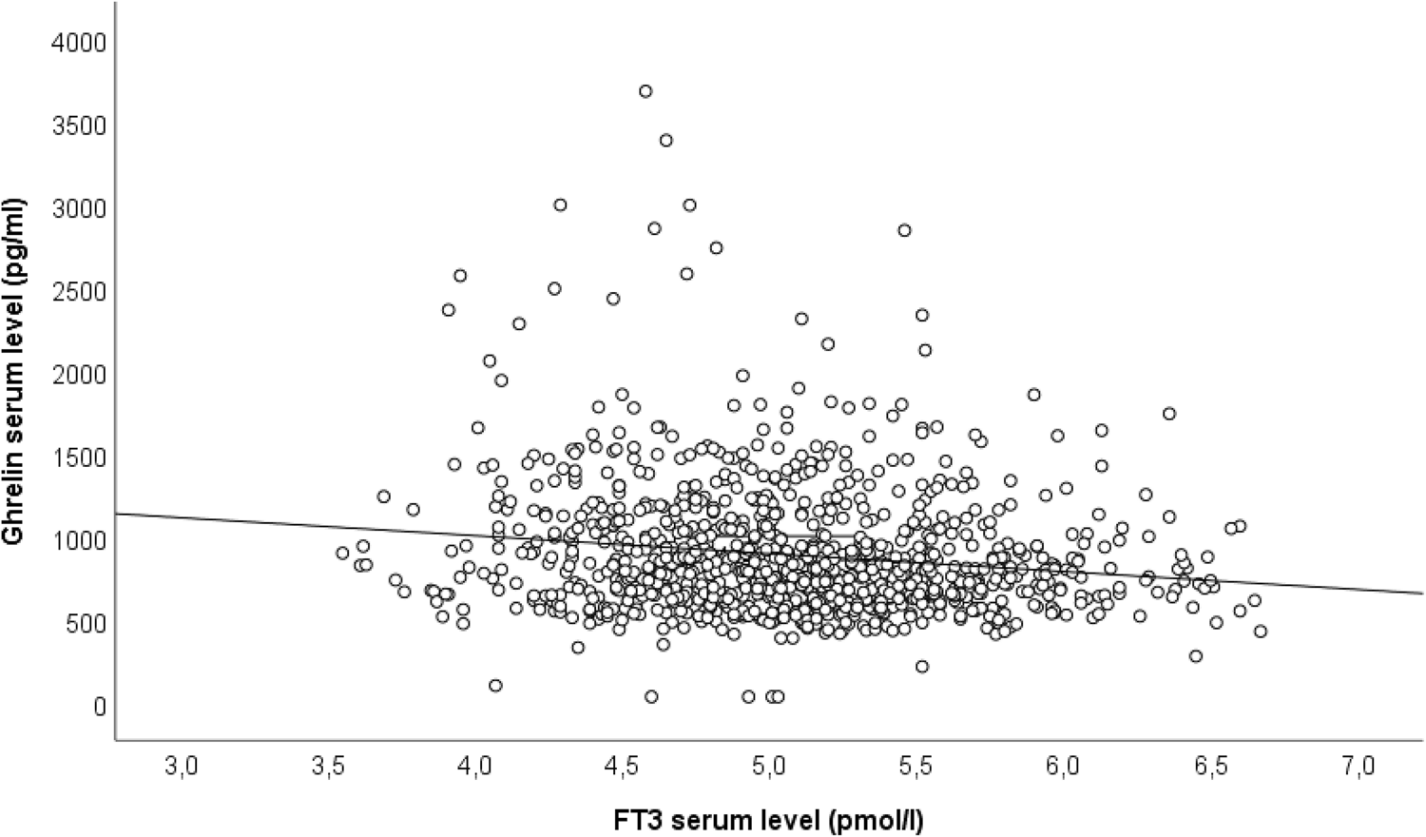
Association between total ghrelin serum and FT3 serum levels, adjusted for BMI, age, alcohol consumption and smoking status

TSH and fT4 levels showed no association with total ghrelin serum levels (adjusted for age, sex, BMI, alcohol consumption, smoking status, fT3-levels and fT4- and TSH levels respectively). fT3 showed a significant negative association with ghrelin serum levels when adjusted for age, sex, BMI, alcohol consumption, smoking status, TSH- and fT4- levels (*ß* = − 0.0001, standardized *β* = − 0.102, 95% CI − 0.0002; − 0.00006; *p* < 0.001) (see also Fig. 1).

This effect was present in both men (*ß* = − 0.0002, standardized *β* = − 0.091; 95% CI − 0.0003; − 0.00003; *p* = 0.013) and women (*ß* = − 0.0001, standardized *β* = − 0.132; 95% CI − 0.0003; − 0.00003; *p* = 0.010), see also Table 2).

**Table 2:** Results of multiple linear regression analyses regarding the association between ghrelin serum levels and TSH, FT3 and FT4 concentrations in euthyreotic individuals

Variables	Regression coefficient *β* (95% CI)	Stan-dardized ß	*t*	*p* value
TSH concentration in the total sample, mU/l (*N* = 1012**)** Corrected *R*^2^ = 0.057; *F* = 8.70; *p* < 0.001***				
Multivariate model^a^	–	–	–	–
Ghrelin serum levels	0.0000051 (− 0.00011; 0.00012)	0.003	0.09	0.93
Age	− 0.011 (− 0.014; − 0.008)	− 0.229	− 6.76	< 0.001***
Sex	0.028 (− 0.077; 0.133)	0.019	0.52	0.60
Alcohol consumption	− 0.001 (− 0.003; 0.002)	− 0.014	− 0.41	0.68
Smoking status	− 0.085 (− 0.147; − 0.024)	− 0.088	− 2.72	0.007**
BMI	0.010 (− 0.001; 0.020)	0.058	1.79	0.08^+^
FT3 concentration	0.046 (− 0.041; 0.134)	0.036	1.04	0.30
FT4 concentration	− 0.049 (− 0.075; − 0.022)	− 0.113	− 3.58	< 0.001***
FT3 concentration in the total sample, mU/l (*N* = 1012) Corrected *R*^2^ = 0.230; *F* = 38.76; *p* < 0.001***				
Multivariate model^a^	–	–	–	–
Ghrelin serum levels	− 0.00015 (− 0.00023; − 0.000063)	− 0.102	− 3.46	< 0.001***
Age	− 0.010 (− 0.012; − 0.008)	− 0.269	− 8.92	< 0.001***
Sex	− 0.308 (− 0.379; − 0.237)	− 0.263	− 8.46	< 0.001***
Alcohol consumption	0.00017 (− 0.002; 0.002)	0.006	0.18	0.85
Smoking status	0.061 (0.018; 0.104)	0.081	2.76	0.006**
BMI	− 0.002 (− 0.010; 0.005)	− 0.018	− 0.61	0.55
TSH concentration	0.023 (− 0.021; 0.067)	0.030	1.04	0.30
FT4 concentration	0.065 (0.046; 0.083)	0.193	6.87	< 0.001***
FT4 concentration in the total sample, mU/l (N = 1012) Corrected *R*^2^ = 0.069; *F* = 10.32; *p* < 0.001***				
Multivariate model^a^	–	–	–	–
Ghrelin serum levels	0.00014 (− 0.00013; 0.00041)	0.033	1.02	0.31
Age	0.003 (− 0.004; 0.011)	0.032	0,93	0,35
Sex	− 0.141 (− 0.383; 0.100)	− 0.041	− 1.15	0.25
Alcohol consumption	− 0.009 (− 0.015; − 0.003)	− 0.101	− 3.07	0.002**
Smoking status	− 0.111 (− 0.253; 0.030)	− 0.050	− 1.54	0.12
BMI	− 0.023 (− 0.047; 0.001)	− 0.060	− 1.86	0.06^+^
TSH concentration	− 0.259 (− 0.401; − 0.117)	− 0.112	− 3.58	< 0.001***
FT3 concentration	0.692 (0.495; 0.890)	0.233	6.87	< 0.001***
TSH concentration in men, mU/l (*n* = 659) Corrected *R*^2^ = 0.054; *F* = 6.37; *p* < 0.001***				
Multivariate model^a^	–	–	–	–
Ghrelin serum levels	− 0.000049 (− 0.00022; 0.000125)	− 0.022	− 0.56	0.58
Age	− 0.011 (− 0.015; − 0.007)	− 0.242	− 5.69	< 0.001***
Alcohol consumption	− 0.001 (− 0.004; 0.002)	− 0.026	− 0.67	0.50
Smoking status	− 0.090 (− 0.164; − 0.017)	− 0.095	− 2.42	0.02*
BMI	0.014 (− 0.000067; 0.028)	0.079	1.95	0.051^+^
FT3 concentration	− 0.019 (− 0.126;0.087)	− 0.015	− 0.35	0.72
FT4 concentration	− 0.039 (− 0.072; − 0.007)	− 0.092	− 2.36	0.02*
FT3 concentration in men, mU/l (*n* = 659) Corrected *R*^2^ = 0.151; *F* = 17.70; *p* < 0.001***				
Multivariate model^a^	–	–	–	–
Ghrelin serum levels	− 0.000159 (− 0.000284; − 0.000034)	− 0.091	− 2.49	0.013*
Age	− 0.011 (− 0.014; − 0.009)	− 0.327	− 8.35	< 0.001***
Alcohol consumption	− 0.000124 (− 0.002; 0.002)	− 0.005	− 0.13	0.90
Smoking status	0.065 (0.012; 0.118)	0.090	2.41	0.02*
BMI	0.004 (− 0.006; 0.014)	0.029	0.76	0.45
TSH concentration	− 0.010 (− 0.066; 0.046)	− 0.013	− 0.35	0.72
FT4 concentration	0.057 (0.034; 0.080)	0.176	4.80	< 0.001***
FT4 concentration in men, mU/l (*n* = 659) Corrected *R*^2^ = 0.063; *F* = 7.36; *p* < 0.001***				
Multivariate model^a^	–	–	–	–
Ghrelin serum levels	0.000195 (− 0.000215; 0.000605)	0.036	0.93	0.35
Age	0.002 (− 0.007; 0.011)	0.018	0.43	0.67
Alcohol consumption	− 0.009 (− 0.015; − 0.003)	− 0.112	− 2.89	0.004**
Smoking status	− 0.040 (− 0.213; 0.133)	− 0.018	− 0.45	0.65
BMI	− 0.044 (− 0.077; − 0.011)	− 0.106	− 2.63	0.009**
TSH concentration	− 0.216 (− 0.397; − 0.036)	− 0.092	− 2.36	0.02*
FT3 concentration	0.602 (0.356; 0.848)	0.194	4.80	< 0.001***
TSH concentration in women, mU/l (*n* = 353) Corrected *R*^2^ = 0.063; *F* = 4.38; *p* < 0.001***				
Multivariate model^a^	–	–	–	–
Ghrelin serum levels	0.000047 (− 0.000116; 0.00021)	0.030	0.57	0.57
Age	− 0.011 (− 0.016; − 0.005)	− 0.212	− 3.70	< 0.001***
Alcohol consumption	0.003 (− 0.006; 0.012)	0.034	0.64	0.52
Smoking status	− 0.082 (− 0.196; 0.031)	− 0.078	− 1.42	0.16
BMI	0.007 (− 0.009; 0.023)	0.047	0.85	0.39
FT3 concentration	0.173 (0.016; 0.329)	0.121	2.17	0.03*
FT4 concentration	− 0.066 (− 0.113; − 0.018)	− 0.146	− 2.73	0.007**
FT3 concentration in women, mU/l (*n* = 353) Corrected *R*^2^ = 0.139; *F* = 9.13; *p* < 0.001***				
Multivariate model^a^	–	–	–	–
Ghrelin serum levels	− 0.000143 (− 0.000251; − 0.000034)	− 0.132	− 2.59	0.010*
Age	− 0.007 (− 0.011; − 0.003)	− 0.206	− 3.75	< 0.001***
Alcohol consumption	0.002 (− 0.004; 0.008)	0.027	0.53	0.60
Smoking status	0.052 (− 0.024; 0.129)	0.071	1.34	0.18
BMI	− 0.010 (− 0.021; 0.001)	-0.094	− 1.77	0.08^+^
TSH concentration	0.078 (0.007; 0.149)	0.111	2.17	0.03*
FT4 concentration	0.081 (0.050; 0.112)	0.258	5.14	< 0.001***
FT4 concentration in women, mU/l (*n* = 353) Corrected *R*^2^ = 0.076; *F* = 5.15; *p* < 0.001***				
Multivariate model^a^	–	–	–	–
Ghrelin serum levels	0.000147 (− 0.000214; 0.000508)	0.043	0.80	0.42
Age	0.007 (− 0.006;0.019)	0.061	1.05	0.30
Alcohol consumption	− 0.005 (− 0.026;0.015)	− 0.028	− 0.53	0.60
Smoking status	− 0.239 (− 0.491;0.013)	− 0.102	− 1.87	0.06^+^
BMI	0.004 (− 0.032; 0.040)	0.012	0.22	0.83
TSH concentration	− 0.322 (− 0.555; − 0.090)	− 0.144	− 2.73	0.007**
FT3 concentration	0.880 (0.544; 1.217)	0.276	5.14	< 0.001***

*b* regression coefficient, *β* standardized regression coefficient, *CI* confidence interval, *N/n* sample sizes

^+^*p* < 0.10; *p < 0.05; **p < 0.01; ***p < 0.001

^a^All multivariate linear regression models have been adjusted for age, sex (in total group analyses only), alcohol consumption, smoking status (1 = active smoking; 0 = non-smoking), BMI scores and other thyroid hormone concentrations

An extreme group comparison revealed significantly lower FT3 levels in individuals from the highest quartile group according to ghrelin serum levels than in those from the lowest quartile group (*Z* = − 4.27; *p* = 0.00002). Corresponding differences in the male subgroup failed to be significant (*Z* = − 1.79; *p* = 0.07). In women, the FT3 levels in subjects from the highest and lowest quartile group according to ghrelin serum levels were nearly identical (mean score: 4.8 pmol/l; *Z* = 0; *p* = 1) (for details see Table 3).

**Table 3:** Extreme-group-comparison of FT3 concentrations in the highest and lowest quartiles of ghrelin serum levels

Quar-tiles	Ghrelin serum levels in pg/ml			Mean FT3 concentrations (SD) (pmol/l)			N		
	All	Men	Wo-men	All	Men	Wo-men	All	Men	Wo-men
Lowest: <	644.25	615	716	5.13 (0.54)	5.21 (0.53)	4.77 (0.45)	253	166	88
Highest: >	1051	932	1245.5	4.93 (0.54)	5.12 (0.54)	4.81 (0.53)	254	168	88
–	–	–	–	*Z* = − 4.27^a^ *p* < 0.001***	*Z* = − 1.79^a^ *p* = 0.073^+^	*Z* = 0^a^ *p* = 1	–	–	–

*SD* standard deviation

^+^p < 0.10; *p < 0.05; **p < 0.01; ***p < 0.001

^a^A Mann–Whitney U test was performed to assess group differences in FT3 concentrations due to non-normal distribution of the dependent variable (Kolmogorov–Smirnov-Test: *p* < 0.05)

## Discussion

In this study, we determined the relationship between total ghrelin serum levels and serum levels of the thyroid hormones TSH, fT4 and fT3 in a large, population-based sample of euthyroid subjects. Ghrelin was negatively associated with serum levels of fT3 and showed no association with levels of TSH and fT4. In an extreme-group comparison, there was a significant difference in fT3 levels between the highest and lowest quartile of serum ghrelin levels. Our findings are in line with studies indicating a negative association between ghrelin and thyroid hormones.

Current data suggests that there is a bidirectional antagonistic effect between ghrelin and thyroid hormones. In hypothyroid rats, ghrelin serum levels were elevated [38, 39]. Furthermore, ghrelin was shown to directly inhibit the production of thyroglobulin [40]. Likewise, hypothyroidism was associated with a higher production of ghrelin mRNA in the stomach mucosa of rats and hyperthyroidism with a lower concentration of mRNA [41]. In human studies, too, the vast majority of studies show higher ghrelin levels in hypothyroidism and lower ghrelin levels in hyperthyroidism (despite the often present increased appetite) [25, 26, 38, 42–44], with some showing a direct negative correlation between fT3 and ghrelin [26, 45] and some showing only group effects [38, 44]. Albeit, these are correlational studies from which no direct causal conclusion can be drawn. However, the few interventional studies show both, that ghrelin affects (predominantly inhibits) the HPT axis [17, 18, 27] and that TSH decreases ghrelin levels [46].

Ghrelin seems to affect the HPT-axis on multiple levels. Intervention studies in animals and humans suggest a suppressive effect of ghrelin at hypothalamic level resulting in decreased TSH levels [17, 18, 27]. The decrease in TSH in the study by Kluge et al. 2010 was accompanied by a subtle increase in fT4, which would be in line with a direct effect of ghrelin on the thyroid gland [47]. Thus, a suppression of TSH might have in part been caused by feedback inhibition of fT4 [18]. However, the authors argue that it is doubtful that the observed decrease in TSH is solely explained by the increase of fT4. Administration of much higher doses of fT4 led to a smaller decrease in TSH in another study [48] and ghrelin acts on hypothalamic neurons that decrease the activity of TRH-neurons [49–52]. These findings suggest a physiological relevance of ghrelin in the regulation of the HPT axis.

Our finding that ghrelin was only associated with fT3, but not fT4 and TSH contrasts to other, smaller studies where, if associations were found, they were between ghrelin and both fT4 and fT3 [25, 26, 38, 42–44]. This might be related to the type of study participants (patients with thyroid dysfunction vs. healthy subjects) and sample size with some studies being too small to unmask an existing association. Furthermore, our results direct the attention to another system regulating thyroid hormone concentration and metabolism, the system of the deiodinases. FT3 is the most biologically active form of thyroid hormones and is produced in the body in mainly two ways [24]: in the thyroid gland, together with fT4 in a ratio of approximately 1:14 [23, 24] and through a system operating at the intracellular level, the iodothyronine deiodinases (DIO) [23]. Three deiodinase enzymes have been identified so far (DIO1, DIO2 and DIO3), which all catalyse the removal of an iodine atom both from fT4 and fT3. DIO2 acts only in an activating manner, converting fT4 to active fT3, while DIO3 actions are exactly opposite, deactivating fT3 and fT4 to diiodothyronine (T2) or reverse T3, respectively, which are both biologically inactive [53]. DIO1 is expressed in the liver and kidney and acts in an activating manner [54]. While the first mechanism accounts for approximately 20% of fT3 plasma levels, the peripheral conversion provides the remaining 80% [55]. The regulation of fT3 and fT4 plasma levels is complex and seems to be an interaction between the action of the HPT-axis, thyroid hormone transporters and deiodinases [23]. The exact contribution of the deiodinase enzymes is debated, especially in humans, where data is very scarce [23]. DIO2 is thought to be the major source of plasma fT3 [23, 56], however, DIO1 is also believed to play a role, due to its location in the plasma membrane and the fact that fT3 produced by DIO1 exits the cell after 30 min compared to 8 h for DIO2 [23, 57]. The contribution of DIO1 might even depend on whether the organism is in a euthyroid or hyperthyroid state [23, 58]. In animals, DIO1 and DIO2 knockout (KO) mice showed fT3 levels within the reference range [59, 60] due to higher TSH levels and higher secretion of fT3 from the thyroid [61]. These results indicate the existence of compensatory mechanisms in the HPT-axis and in iodothyronine degradation, but also that the contribution of the thyroid gland to fT3 levels might be more pronounced in animals than in humans [23, 62, 63].

The deiodinase system allows the organism to modify the intracellular concentration of fT3 at a single-cell level, depending on the current demand [23]. Under fasting conditions, the conversion of fT4 to fT3 is reduced, for example by decreased expression of DIO1 in the liver and kidney [54, 64]. In addition, the rate of degradation of rT3 seems to be reduced [54, 65], most likely as an energy-saving mechanism. Leptin seems to play a major role in this adaption [54] and it affects the deiodinase system in a stimulatory manner [66]. These are results from animal studies and might not exactly reflect the situation in humans.

Surprisingly, to our knowledge, so far no data exists investigating the relationship between ghrelin and the deiodinase system. While our correlational data does not allow for causal conclusions, it is an intriguing and biologically plausible thought that ghrelin might affect the activity of the deiodinase system as a further mechanism to reduce energy expenditure in fasting conditions. Assuming this is the case, then an inhibitory effect of ghrelin on DIO1 and DIO2 would seem plausible. Further research is needed to investigating the nature of the relationship between ghrelin and the DIO-system. This could lead to a more nuanced understanding of ghrelin’s role in regulating metabolic adaption to hypocaloric and fasting states.

As outlined above, it is well documented that the HPT-axis adapts to fasting states. Fasting leads to decreased serum levels of fT3 after 24 h fasts by about 6% in humans [67], while ghrelin levels rise in fasting states [5]. However, this decrease in fT3 is accompanied by a more pronounced decrease in serum TSH levels by about 40% in the study by Basolo et al. and in previous studies [54, 67]. We did not, however, find an association of ghrelin levels and TSH. Furthermore, subjects in our study fasted merely overnight, instead of 24 h. It is thus unlikely that the association between fT3 and ghrelin merely represents an unrelated fasting response.

Main limitation of the presented study is the cross-sectional design, not allowing to draw causal conclusions. Furthermore, only total ghrelin but not acylated ghrelin was determined and samples were not acidified or pre-treated with protease inhibitors. The main strength of the study is the large sample size, which is a unique feature of this report. In addition, the study design and data collection were highly standardized.

## Conclusion

This is to date the largest association study of total ghrelin serum levels and the thyroid hormones TSH, fT3 and fT4 in euthyroid subjects. The negative association of ghrelin serum levels with fT3, but not with fT4 and TSH, might be an initial indicator that ghrelin acts on the system of the deiodinases. Our results should initiate further research to facilitate a better understanding of the metabolic effects of ghrelin and its interaction with the thyroid system.

## Supplementary Information

Below is the link to the electronic supplementary material.

Supplementary file 1 (DOCX 13 KB)

## Data Availability

The data that support the findings of this study are available from the Leipzig Research Center for Civilisation Diseases (LIFE) but restrictions apply to the availability of these data, which were used under license (project number: PV_0358_Kluge) for the current study, and so are not publicly available. Data are, however, available from the authors upon reasonable request and with permission of LIFE.
