# Supplementary material for: Free triiodothyronine (T3) is negatively associated with fasting ghrelin serum levels in a population sample of euthyroid subjects
Source: J Endocrinol Invest. 2021 Apr 21;44(12):2655–64. doi: 10.1007/s40618-021-01578-5 (PMC8572188; doi:10.1007/s40618-021-01578-5)
Supplement: Supplementary file 1 — (DOCX 13 KB) [file 40618_2021_1578_MOESM1_ESM.docx]

|  | Definition | n |
| --- | --- | --- |
| Overt hypothyroidism | TSH >3.77 mU/l AND fT4 <12.8 pmol/l AND/OR fT3 <3.1 pmol/l | 0 |
| Subclinical hypothyroidism | TSH >3.77 mU/l AND fT4 12.8-20.4 pmol/l AND fT3 3.1-6.79 pmol/l | 49 |
| Overt hyperthyroidism | TSH <0.4 mU/l AND fT4 >20.4 pmol/l AND/OR fT3 >6.79 pmol/l | 2 |
| Subclinical hyperthyroidism | TSH <0.4 mU/l AND fT4 12.8-20.4 pmol/l AND fT3 3.1-6.79 pmol/l | 34 |
| Elevated fT4 with normal TSH | TSH 0.4-3.77 mU/l, fT4>20.4 pmol/l | 93 |
| Reduced fT4 with normal TSH | TSH 0.4-3.77 mU/l, fT4 <12.8 pmol/l | 65 |
| Elevated fT3 with normal TSH | TSH 0.4-3.77 mU/l, fT3> 6.79 pmol/l | 14 |
| Reduced fT3 with normal TSH | TSH 0.4-3.77 mU/l, fT3 <3.1 pmol/l | 3 |
| Other constellations | | 35 |
| Total |  | 295 |

**Supplementary Table 1:** Constellation of thyroid hormones in subjects excluded from the analysis
